# Tuberculosis in Pregnancy: A Review

**DOI:** 10.1155/2012/379271

**Published:** 2011-11-01

**Authors:** Olabisi M. Loto, Ibraheem Awowole

**Affiliations:** Department of Obstetrics and Gynaecology, Obafemi Awolowo University Teaching Hospital, P.M.B. 5538, Ile-Ife, Osun State, Nigeria

## Abstract

Tuberculosis (TB) was declared a public health emergency by WHO in 2005. The disease is a significant contributor to maternal mortality and is among the three leading causes of death among women aged 15–45 years in high burden areas. The exact incidence of tuberculosis in pregnancy, though not readily available, is expected to be as high as in the general population. Diagnosis of tuberculosis in pregnancy may be challenging, as the symptoms may initially be ascribed to the pregnancy, and the normal weight gain in pregnancy may temporarily mask the associated weight loss. Obstetric complications of TB include spontaneous abortion, small for date uterus, preterm labour, low birth weight, and increased neonatal mortality. Congenital TB though rare, is associated with high perinatal mortality. Rifampicin, INH and Ethambutol are the first line drugs while Pyrazinamide use in pregnancy is gaining popularity. Isoniazid preventive therapy is a WHO innovation aimed at reducing the infection in HIV positive pregnant women. Babies born to this mother should be commenced on INH prophylaxis for six months, after which they are vaccinated with BCG if they test negative. Successful control of TB demands improved living conditions, public enlightenment, primary prevention of HIV/AIDS and BCG vaccination.

## 1. Introduction

Tuberculosis (TB) is believed to be nearly as old as human history. Traces of it in Egyptian mummies date back to about 7000 years ago, when it was described as phthisis by Hippocrates [1]. It was declared a public health emergency in the African Region in 2005 [1] and has since continued to be a major cause of disability and death. About 9.4 million new cases of tuberculosis were diagnosed in 2009 alone and 1.7 million people reportedly died from the disease in the same year, translating to about 4700 deaths per day [2]. 

About one-third of the world's population (estimated to be about 1.75 billion) is infected with the tubercule bacillus [3]. As much as 75% of individuals with TB are within the economically productive age group of 15 to 54 years. This significantly impairs socioeconomic development, thereby perpetuating the poverty cycle [4]. 

Tuberculosis has been on the rise in tandem with HIV/AIDS. This is because people with HIV/AIDS, whose immune systems are weakened have with a 20–37 times the risk of developing a progressive disease compared with HIV-negative individuals [4]. 

## 2. Microbiology of Tuberculosis

*Mycobacterium tuberculosis, *an aerobic, non-spore-forming, nonmotile bacillus, is one of five members of the Mycobacterium tuberculosis complex, others being* M. bovis, M. ulcerans, M. Africanum, and M. microti,* though M. tuberculosis is the major human pathogen. It belongs to the family Mycobacteriaceae. Other *Mycobacterium* species that may infect humans include *Mycobacterium leprae*, *M. avium*, *M. Intracellulare,* and *M. scrofulaceum.*

## 3. Pathophysiology

Tuberculosis affects almost every organ in the body, but the usual site of the disease is the lungs, accounting for more than 80 percent of tuberculosis cases [5]. The pattern of the infection in HIV positive patients may, however, be different, with increasing trends towards extrapulmonary spread [6].

Almost all tuberculosis infections are caused by inhalation of infectious particles aerosolized by coughing, sneezing, talking, or manipulation of infected tissues. Other modalities of transmission may, however, include ingestion of unpasteurised milk and direct implantation through skin abrasion or the conjunctiva. Aerosolized tuberculosis particles with sizes ranging between 1 and 5 um are carried to the terminal air spaces of high-airflow areas, where multiplication of the tubercle occurs. Following phagocytosis by pulmonary macrophages, a granulomatous reaction may be initiated, in conjunction with the regional lymph nodes, thereby forming the Ghon's focus. The bacilli remain in a state of dormancy within the Ghon's focus, from where they may later become reactivated.

## 4. Tuberculosis in Pregnancy

The wide array of opinion of Medical practitioners on tuberculosis in pregnancy simply reflects the Public Health significance of the condition. It is best described as a doubled-edged sword, one blade being the effect of tuberculosis on pregnancy and the pattern of growth of the newborn, while the other is the effect of pregnancy on the progression of tuberculosis. 

Tuberculosis not only accounts for a significant proportion of the global burden of disease, it is also a significant contributor to maternal mortality, with the disease being among the three leading causes of death among women aged 15–45 years [2].

The exact incidence of tuberculosis in pregnancy is not readily available in many countries due to a lot of confounding factors. It is, however, expected that the incidence of tuberculosis among pregnant women would be as high as in the general population, with possibly higher incidence in developing countries. 

Earlier study by Schaefer reported a new case rate of 18–29/100,000 in pregnancy, which was similar to the 19–39/100,000 reported for the city of New York [7]. A recent United Kingdom study, however, quoted an incidence of 4.2 per 100,000 maternities [8], which may be a reflection the current global fall in the incidence of the disease [2].

## 5. Effects of Pregnancy on Tuberculosis

Researchers from the days of Hippocrates have expressed their worries about the untoward effects that pregnancy may have on preexisting tuberculosis. Pulmonary cavities resulting from tuberculosis were believed to collapse as a result of the increased intra-abdominal pressure associated with pregnancy. This belief was widely held till the beginning of the fourteenth century! Indeed, a German physician recommended that young women with TB should get married and become pregnant to slow the progression of the disease. This was practiced in many areas till the 19th century [9], while in the early 20th century, induced abortion was recommended for these women [10, 11]. Researchers like Hedvall [12] and Schaefer [7], however, demonstrated no net benefit or adverse effect of pregnancy on the progression of TB. Frequent, consecutive pregnancies may, however, have a negative effect, as they may promote recrudescence or reactivation of latent tuberculosis.

It is, however, important to note that the diagnosis of tuberculosis in pregnancy may be more challenging, as the symptoms may initially be ascribed to the pregnancy. The weight loss associated with the disease may also be temporarily masked by the normal weight gain in pregnancy. 

## 6. Effects of Tuberculosis on Pregnancy

The effects of TB on pregnancy may be influenced by many factors, including the severity of the disease, how advanced the pregnancy has gone at the time of diagnosis, the presence of extrapulmonary spread, and HIV coinfection and the treatment instituted. 

The worst prognosis is recorded in women in whom a diagnosis of advanced disease is made in the puerperium as well as those with HIV coinfection. Failure to comply with treatment also worsens the prognosis [13].

Other obstetric complications that have been reported in these women include a higher rate of spontaneous abortion, small for date uterus, and suboptimal weight gain in pregnancy [14, 15]. Others include preterm labour, low birth weight and increased neonatal mortality [13]. Late diagnosis is an independent factor, which may increase obstetric morbidity about fourfolds, while the risk of preterm labour may be increased ninefolds [15–18].

## 7. Tuberculosis and the Newborn

Congenital tuberculosis is a rare complication of *in utero *tuberculosis infection [19] while the risk of postnatal transmission is significantly higher [20]. Congenital tuberculosis may be as a result of haematogenous spread through the umbilical vein to the foetal liver or by ingestion and aspiration of infected amniotic fluid [21]. A primary focus subsequently develops in the liver, with involvement of the peri-portal lymph nodes. The tubercle bacilli infect the lungs secondarily, unlike in adults where over 80% of the primary infections occur in the lungs [5]. 

Congenital tuberculosis may be difficult to distinguish from other neonatal or congenital infections from which similar symptoms may arise in the second to the third week of life. These symptoms include hepato-splenomegaly, respiratory distress, fever, and lymphadenopathy. Radiographic abnormalities may also be present but these generally appear later [13]. The diagnosis of neonatal tuberculosis may, however, be facilitated by employing a set of diagnostic criteria developed by Cantwell et al. [22], including the demonstration of primary hepatic complex/caseating granuloma on percutaneous liver biopsy at birth, tuberculous infection of the placenta, or maternal genital tract tuberculosis, and the demonstration of lesions during the first week of life. The possibility of postnatal transmission must be excluded by a thorough investigation of all contacts, including hospital staffs and attendants.

As much as half of the neonates delivered with congenital tuberculosis may eventually die, especially in the absence of treatment [7, 23–26].

## 8. Diagnosis of Tuberculosis in Pregnancy

To diagnose this condition, history of exposure to individuals with chronic cough or recent visit to areas endemic with tuberculosis should be obtained. History of symptoms, which is likely to be the same as in nonpregnant women, is also essential. Caution must, however, be exercised, as these symptoms may be nonspecific in pregnancy [27, 28]. These symptoms include night sweat, evening pyrexia, haemoptysis, progressive weight loss, and chronic cough of over 3 weeks duration. There may also be a history of ineffective attempts at antibiotics therapy [27, 29].

In pregnant women with suggestive symptoms and signs of TB, a tuberculin skin test should be carried out. This has since been accepted to be safe in pregnancy [21, 30]. The debate, however, is about the sensitivity of tuberculin test during pregnancy. Earlier reports suggested diminished tuberculin sensitivity in pregnancy [31], while recent studies revealed no significant differences in the pregnant and nonpregnant populations [27, 32–35].

The two types of tuberculin skin tests are discussed below.

### 8.1. Tine Test

This test utilises an instrument with multiple needles that are dipped in a purified form of the TB bacteria called old tuberculin (OT). The skin is pricked with these needles and the reaction is analysed 48–72 hours later. It is, however, no longer popular except in large population screening.

### 8.2. Mantoux Test

 A single-needle intradermal injection of 0.1 mL of purified protein derivative (5 Tuberculin units) is administered, and the skin reaction is analysed 48–72 hours later, based on the largest diameter of the indurations developed. It is a more accurate and reproducible test than the Tines test. 

False-positive results may be obtained in individuals who had previously been vaccinated with the BCG vaccine, those with previously treated tuberculosis, as well as in people with infection from other Mycobacterium species. False negatives on the other hand are commonly due to a compromised immune system and technical errors [36].

A chest radiograph with abdominal lead shield may be done after the tuberculin skin testing, though pregnant women are more likely to experience a delay in obtaining a chest X-ray due to concerns about fetal health [27].

Microscopic examination of sputum or other specimen for Acid-fast bacilli (AFB) remains the cornerstone of laboratory diagnosis of TB in pregnancy. Three samples of sputum should be submitted for smear, culture, and drug-susceptibility testing. Staining for AFB is also done, using the Ziehl-Neelsen, fluorescent, Auramine-Rhodamine, and the Kinyoun techniques [37]. Light-emitting diode (LED) fluorescent microscopy has recently been introduced to improve diagnosis [37]. According to the WHO's 2009 report on global TB control, the percentage of new cases of smear-positive TB detected ranged between 56 and 68%. The staining techniques may, therefore, not sufficient for the diagnosis of TB, as smear-negative cases will be missed [37].

## 9. Culture

The traditional culture on Lowenstein-Jensen's medium may take 4–6 weeks to obtain a result. This may, however, still be useful in cases of diagnostic doubts and management of suspected drug-resistant tuberculosis [38]. Newer diagnostic tools are now available to facilitate diagnosis, including the liquid Bactec culture medium, which has been endorsed by WHO. Other culture media that could be used include the modified Lowenstein's medium, Petragnani medium, Trudeau Committee medium, Peizer's medium, Dubos Middlebrook media, Tarshis blood agar, Middlebrook's 7-H3, Middlebrook's 7-H9, and Middlebrook's 7H-10 media [38]. Liquidisation and decontamination with N-Acetytl-L-Cysteine in 1% Sodium Hydroxide solution before inoculation may enhance sensitivity [38].

*M. tuberculosis* produces niacin and heat-sensitive catalase and it lacks pigment. It may, therefore, be differentiated from other mycobacterium species using these features. Others include reduction of nitrates and its isoniazide sensitivity, which may, however, not be reliable in cases of INH resistance. 

Molecular Line Probe Assay (LPA) as well as the use of polymerase chain reaction (PCR) are presently facilitating the specific identification of the tubercle bacilli [37].

## 10. Treatment of Tuberculosis

*“Untreated tuberculosis represents a far greater hazard to a pregnant woman and her fetus than does treatment of the disease”* [39]. 

The management of tuberculosis in pregnancy is a multidisciplinary approach, with the team comprising the obstetrician, communicable disease specialty personnel, neonatologists, counselling unit, and public health officials.

Treatment is achieved through the use of Directly Observed Therapy, Short Course (DOTS). This therapy entails the use of combination therapy for at least 6 months, depending on the combination of antituberculous agents that are available. This combination includes isoniazide and rifampicin compulsorily, supported by ethambutol and pyrazinamide [40–44]. 

For patients with drug-susceptible TB and good drug adherence, these regimens will cure around 90% of TB cases. Treatment is done on out-patient basis, unless otherwise indicated [37].

The use of these first-line antituberculous drugs in pregnancy are considered safe for the mother and the baby by The British Thoracic Society, International Union Against Tuberculosis and Lung Disease, and the World Health Organisation [16, 45].

### 10.1. Isoniazide

INH is safe during pregnancy even in the first trimester, though it can cross the placenta [11]. The women must, however, be followed up because of the possibility of INH-induced hepatotoxicity. Pyridoxine supplementation is recommended for all pregnant women taking INH at a dose of 50 mg daily [39, 46].

### 10.2. Rifampicin

This is also believed to be safe in pregnancy, though in an unknown proportion of cases, there may be an increased risk of haemorrhagic disorders in the newborn (some authorities prescribe supplemental vitamin K (10 mg/day) for the last four to eight weeks of pregnancy.) while some other researchers reported the possibility of limb deformity but none of these are in excess of what is obtained in the normal population.

### 10.3. Ethambutol

The retrobulbar neuritis that may complicate the use of this drug in adults generated the fear that it may interfere with ophthalmological development when used in pregnancy but this has not been demonstrated when the standard dose is used. This was also confirmed in experimental studies on some abortuses [47].

### 10.4. Pyrazinamide

The use of pyrazinamide in pregnancy was avoided by many physicians for a long time due to unavailability of adequate data on its teratogenicity. Presently, many international organizations now recommend its use, including the International Union Against Tuberculosis And Lung diseases (IUATLD), British Thoracic Society, American Thoracic Society, the World Health Organisation as well as the Revised National Tuberculosis Control Programme of India. There are no reports of significant adverse events from the use of this drug in the treatment of TB in pregnant women despite its use as part of the standard regimen in many countries [48]. 

Its use is particularly indicated in women with tuberculous meningitis in pregnancy, HIV coinfection, and suspected INH resistance [49–52]. Breastfed infants of mothers on antituberculous therapy should, however, be monitored for jaundice, which may suggest drug-induced hepatitis, as well as joint pains resulting from drug-induced hyperuricaemia.

### 10.5. Streptomycin

The drug has been proven to be potentially teratogenic throughout pregnancy. It causes fetal malformations and eighth-nerve paralysis, with deficits ranging from mild hearing loss to bilateral deafness. Many centres are against the use of this drug in pregnancy [49, 53, 54].

## 11. Multidrug-Resistant Tuberculosis in Pregnancy (MDR-TB)

Pregnant women with MDR-TB have a less favourable prognosis [55]. They may sometimes require treatment with second-line drugs, including cycloserine, ofloxacin, amikacin, kanamycin, capreomycin, and ethionamide. The safety of these drugs is unfortunately not well-established in pregnancy [49]. 

Para-amino salicylic acid had been used as combination therapy with INH in pregnancy in the past without any significant teratogenic side effects, though maternal gastrointestinal side effects may be pronounced. 

Ethionamide is associated with growth retardation, central nervous system and skeletal abnormalities in animal studies involving rats and rabbits [56, 57]. Human studies also demonstrated increased central nervous system defects following its use in early pregnancy [58]. Its use is, therefore, not recommended in pregnancy.

Therapeutic abortion has been proposed as an option of management for these women [59], as MDR-TB poses more risk to the woman and the society at large. Another option is to delay initiating treatment to the second trimester where possible [10]. Individualised Treatment Regimen (ITR) using various combinations of the 2nd line antituberculous agents based on their susceptibility profile had, however, been tried in some pregnant women with no adverse obstetric outcome [60].

The outlook for those patients is expected to improve as experience and knowledge in the management of the condition increases.

## 12. Treatment of TB in Lactating Women

Breastfeeding is simply the cheapest and healthiest way to feed a baby. The final decision on breastfeed must, therefore, be taken with necessary input from the neonatologists, obstetricians, and pharmacologists. The American Academy of Pediatrics recommends that women with tuberculosis who have been treated appropriately for two weeks or more and who are not considered contagious may breastfeed [61], while the RNTCP recommends breast-feeding of neonates regardless of the mother's TB status [62].

 Antituberculous drugs are excreted into breast milk, though the dose is less compared with the therapeutic dose for infants. Breastfed infants may receive as much as 20% of the therapeutic dose of INH for infants, while other antituberculous drugs are less excreted. No toxicity has been reported from this small concentration in breast milk [49]. Caution must, however, be exercised as the breast milk dose may contribute to the development of abnormally high plasma levels in newborns who are on antituberculous medications. To minimise this possibility, the mother may take her medications immediately after a feed and substitute a bottle for the next feed. She may then return to her usual pattern of feeding [49, 63].

Pyridoxine deficiency may cause seizures in the newborn. Supplemental pyridoxine should, therefore, be administered to infants on INH or whose mother is taking the drug. 

Breastfeeding may be discouraged in women who are yet to commence treatment at the time of delivery and those who are still actively excreting the bacillus while coughing. It may also be discouraged as part of a prevention of mother to child transmission in HIV coinfection and women with tuberculosis of the lactiferous ducts or glands.

 In the absence of evidence of congenital tuberculosis, isoniazide (10 mg/kg/day) should be commenced at birth and continued for six months. Clinical or radiological features of active tuberculosis and a positive tuberculin skin test are indications for a full course of anti-tuberculous treatment. The tuberculin skin test and chest X-rays are done at 6 weeks, 12 weeks, and 6 months. The baby is vaccinated with BCG at 6 months if these tests are negative. The baby is, however, changed to multiple drug therapy if any of these tests turn positive during the period of monitoring.

## 13. HIV and TB Coinfection in Pregnancy

HIV and TB are inextricably linked. Their effect is even more deadly in pregnancy, when they may contribute significantly to maternal morbidity and mortality. Over 50% of the maternal mortality occurring in mothers with TB in pregnancy is due to coinfection with HIV [64]. Moreover, treatment is complicated by the challenges of adherence, polypharmacy and the overlapping side effect profiles of antituberculosis and antiretroviral drugs [65–67]. 

The key concern is about the interactions between the rifamycins and antituberculous drugs. The suboptimal outcomes of therapeutic trials without a rifamycin has made the use of the drug mandatory, even in the face of drug interactions [68, 69].

The spectrum of antiretroviral drugs available for use in pregnancy is limited. Efavirenz is contraindicated before the thirteenth week of gestation, while the risk of toxicity from the use of didanosine and stavudine is significantly increased in pregnancy. Rifampicin may cause a reduction in the serum concentration of efavirenz, though, increasing the dose of efavirenz does not result in any significant outcome [70]. 

Nevirapine, which is an alternative to the use of efavirenz, also exhibits some drug interaction with rifampicin. Rifampicin may lead to the reduction of serum concentration of nevirapine by as much as 50%. To circumvent this problem, rifabutin, another rifamycin that is as effective as rifampicin in the treatment of tuberculosis may be used, as the drug has less effect on the CYP3A system that metabolizes nevirapine [71].

Generally, there is a dearth of studies and data on how pregnancy may affect the aforementioned interactions. Caution is, therefore, of great importance when managing pregnant women with this cruel duo.

## 14. Prevention of Tuberculosis

The BCG vaccine has been incorporated into the National immunization policy of many countries, especially the high burden countries, thereby conferring active immunity from childhood. Nonimmune women travelling to tuberculosis endemic countries should also be vaccinated. It must, however, be noted that the vaccine is contraindicated in pregnancy [72].

The prevention, however, goes beyond this as it is essentially a disease of poverty. Improved living condition is, therefore, encouraged with good ventilation, while overcrowding should be avoided. Improvement in nutritional status is another important aspect of the prevention.

Pregnant women living with HIV are at higher risk for TB, which can adversely influence maternal and perinatal outcomes [73]. As much as 1.1 million people were diagnosed with the co-infection in 2009 alone [2]. Primary prevention of HIV/AIDS is, therefore, another major step in the prevention of tuberculosis in pregnancy. Screening of all pregnant women living with HIV for active tuberculosis is recommended even in the absence of overt clinical signs of the disease.

 Isoniazid preventive therapy (IPT) is another innovation of the World Health Organisation that is aimed at reducing the infection in HIV positive pregnant women based on evidence and experience and it has been concluded that pregnancy should not be a contraindication to receiving IPT. However, patient's individualisation and rational clinical judgement is required for decisions such as the best time to provide IPT to pregnant women [73]. 

Most importantly, governments commitments are highly encouraged so that the World Health Organisation and all other international bodies involved in fighting tuberculosis may succeed in chasing this monster out of all communities.
